# From Targets to Action: Rolling up our Sleeves after Paris

**DOI:** 10.1002/gch2.201600007

**Published:** 2017-01-30

**Authors:** Brigitte Knopf, Sabine Fuss, Gerrit Hansen, Felix Creutzig, Jan Minx, Ottmar Edenhofer

**Affiliations:** ^1^ Mercator Research Institute on Global Commons and Climate Change Torgauer Straße 12–15 10829 Berlin Germany; ^2^ Potsdam Institute for Climate Change Impact Research Telegrafenberg 31 14473 Potsdam Germany; ^3^ Hertie School of Governance Friedrichstraße 180 10117 Berlin Germany; ^4^ Technical University Berlin Chair Economics of Climate Change Straße des 17. Juni 152 10623 Berlin Germany

**Keywords:** Paris Agreement, 1.5°C target, negative emissions, Sustainable infrastructure investments, policy instruments, climate change, decarbonisation

## Abstract

At the United Nations Climate Change Conference in Paris in 2015 ambitious targets for responding to the threat of climate change have been set: limiting global temperature increase to “well below 2 °C […] and to pursue efforts to limit the temperature increase to 1.5 °C”. However, calculating the CO_2_ budget for 1.5 °C, it becomes clear that there is nearly no room left for future emissions. Scenarios suggest that negative emission technologies will play an even more important role for 1.5 °C than they already play for 2 °C. Especially against this background the feasibility of the target(s) is hotly debated, but this debate does not initiate the next steps that are urgently needed. Already the negotiations have featured the move from targets to implementation which is needed in the coming decade. Most importantly, there is an urgent need to develop and implement instruments that incentivize the rapid decarbonization. Moreover, it needs to be worked out how to link the climate and development agenda and prevent a buildup of coal power causing lock‐in effects. Short term entry points into climate policy should now be in the focus instead of the fruitless debate on the feasibility of targets.

## 1 Introduction

The “Paris Agreement” took effect in November 2016, less than a year after the landmark deal was reached at the United Nations (UN) Climate Change Conference in Paris in 2015. The target of limiting global temperature increase to “well below 2 °C […] and to pursue efforts to limit the temperature increase to 1.5 °C above preindustrial levels”^1^ is ambitious. Greenhouse gas emissions will soon need to approach zero to ensure that warming stays below 1.5 °C, unless so‐called negative emission technologies that withdraw carbon from the atmosphere are widely deployed. Unsurprisingly, the feasibility of the 1.5 °C target is a contentious issue at the interface between science and policy.^2^ It distracts from the core challenge which requires policy action, rather than targets, to take center stage. Otherwise, the door to ambitious climate change mitigation rapidly closes.

Although there has long been a call for a 1.5 °C safeguard, especially from vulnerable small island states and developing countries,^3^, ^4^ its inclusion in the UN Paris agreement came as surprise to many, given the heated debate about the feasibility of the 2 °C target in the run‐up to the meeting.^5^, ^6^, ^7^, ^8^ To support its intent, the United Nations Framework Convention on Climate Change (UNFCCC) has asked the Intergovernmental Panel on Climate Change (IPCC) to produce a Special Report on “the impacts of global warming of 1.5 °C above preindustrial levels and related global greenhouse gas emission pathways” by 2018.^1^

In terms of climate impacts, there is little doubt that 1.5 °C would be a more desirable target than 2 °C, as it would limit long‐term sea level rise and the risk of crossing unknown climate‐related thresholds. Some impacts, such as decreasing crop productivity and water availability, threaten to be substantial even at 1.5 °C warming.^9^ Additionally, for some low‐lying areas and sensitive ecosystems, limiting the global temperature increase to 1.5 °C may be their last chance of survival.^3^ However, there are risks and trade‐offs with other sustainability objectives inherent in the mitigation technologies required to meet the target. Examples include the effects of large scale deployment of bioenergy and the conflict with food production, or nuclear power causing severe environmental accidents.^10^, ^11^ The investigation and realization of definitive and desirable action in the short‐term deserves priority.

## 2 The Biophysical Budget Constraint

**Figure 1:**
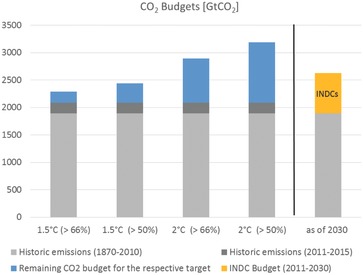
Historic emissions (1870–2010 and 2011–2015) and the total remaining CO_2_ budget (2016 onward) for different likelihoods of staying below 1.5 and 2 °C within the 21st century. For comparison, the cumulative budget absorbed by the Intended Nationally Determined Contributions (INDCs) up to 2030 is given. Source: Historic emissions: IPCC^44^ and Le Quéré et al.;^43^ Budget: IPCC;^12^ INDCs: UNFCCC.^14^ Figure: own representation.

Climate models indicate that the relationship between a temperature target and the residual carbon capacity of the atmosphere (carbon budget) is roughly linear.^12^ **Figure** **1** shows these budgets, compared to historical emissions, for different likelihoods of achieving the 1.5 or 2 °C targets. It demonstrates that, in order to have a likely chance (>66%) of staying below 1.5 °C, a total of only 200 GtCO_2_ can be released from 2016 onward.^12^, ^13^ This exactly represents the emissions of the period 2011–2015, and means that at current rates, the carbon budget for 1.5 °C will be exhausted in five years. It seems likely that to achieve the 1.5 °C target, almost all CO_2_ emissions currently being released will need to be removed from the atmosphere in the future. This implies that wind and solar energy alone will not be enough, as at best, these technologies can reach zero emissions.

Achieving the 2 °C target with a likely chance is somewhat less demanding; the remaining budget of 800 GtCO_2_ allows the energy system to be transformed without relying on large‐scale negative emissions. However, Figure 1 shows that a large proportion of the CO_2_ budget would be absorbed by 2030 if each nation implemented its plans, as outlined in the Intended National Determined Contributions (INDCs) presented in Paris.^14^ Unless the INDCs are tightened, large volumes of emissions will also need to be eliminated by carbon dioxide removal (CDR) technologies. This simple budget calculation highlights that political action contradicts political ambition. This is true for the 2 °C and, in particular, the 1.5 °C target.

## 3 Transformation Requirements

While negative emissions are important for the 2 °C target, for 1.5 °C they become indispensable.^15^ Such negative emissions can be achieved either by combining low‐carbon bioenergy generation with carbon capture and storage (BECCS) or through net land‐use changes.^16^ BECCS in particular, with its large‐scale application of bioenergy, has a considerable land footprint; taking the median amount of BECCS used in IPCC 2 °C scenarios, Smith et al.^17^ estimate that 380–700 Mha would be needed to cultivate the biomass needed. This requires trade‐offs with, and risks to other land‐based activities (e.g., ref. 18, 19, 20, 21 22). Smith et al.^17^ compare BECCS to other CDR technologies such as Direct Air Capture and Enhanced Weathering and find that all conceivable options experience drawbacks in terms of land, energy, or costs. Given the CO_2_ budget constraint, these technologies will all need to be considered in the overall mitigation strategy.

By establishing the 1.5 °C goal, policymakers have bet on the large‐scale availability of negative emissions technologies that could lead to substantial trade‐offs between climate change mitigation and other sustainable development goals.^23^ The more the action to achieve this goal is delayed, the more the reliance on negative emissions to achieving it increases.

However, the debate around negative emissions is futile if the more obvious measures are not implemented first, as it might distract from other important technological requirements for the zero‐carbon transformation. The IPCC clarified that transformation pathways consistent with 2 °C warming rely on both negative emissions and on unprecedented implementation rates of low‐carbon technologies, such as renewables and nuclear energy. They are also characterized by substantial improvements in energy efficiency.^18^ All these requirements are particularly crucial for the 1.5 °C target, as the tiny remaining carbon budget leaves no room to further delay strong global climate policy, abstaining from some mitigation technologies or continue development with high energy demand.^15^, ^18^, ^24^

## 4 Political Feasibility and Ways Forward

Technoeconomic scenarios on climate mitigation clearly demonstrate the need for rapid decarbonization, but lack plausible political narratives.^25^ They remain mostly silent on policy instruments and on the political and distributional implications between and within countries related to such a fundamental transformation of the world economy. The challenge now lies in finding ways to bridge the gap between political ambition and political action. The two most important issues that need to be addressed by both research and policymakers are: (i) ways to foster investment in sustainable infrastructure to avoid a lock‐in to emission intensive infrastructure, especially coal power; and (ii) the development of sufficient and implementable transformative policy instruments.

### 4.1 Sustainable Infrastructure Investments

Infrastructure choices made today will determine carbon emissions in the future. The continued use of existing and new infrastructure as currently planned, contrasts dramatically with climate goals (e.g., ref. 26, 27). One of the most prominent and crucial examples is that of coal‐fired power plants. Coal is currently so cheap that it has, again, become the most important source of energy‐related emissions on the global scale.^28^ Coal resources and reserves are abundant^29^ and the world is experiencing a new buildup of coal in many emerging economies.^28^, ^30^ Once coal power plants are built, there is a considerable lock‐in to carbon‐intensive infrastructure that could inevitably consume large parts of the remaining CO_2_ budget.^31^ Introducing a price on CO_2_ emissions could be an important contribution in determining the correct relative price of coal and with it, avoiding lock‐in. However, in developing countries, economic growth is the key to bringing people out of poverty, and some governments, such as those in India, Vietnam, and South Africa, rely on coal for growth. Therefore, the detrimental effect of carbon prices on poor households needs to be understood. One proposal is to use the revenues generated from carbon pricing to either reduce other taxes,^32^ or invest in infrastructure for the provision of basic needs such as access to water or sanitation.^33^ Future research will be required to explore the opportunities – and barriers – for each country to the implementation of carbon pricing.

### 4.2 Transformative Policy Instruments and Energy Demand Options

Transitions to low‐carbon economies can be achieved by applying different energy supply policies. These include putting a price on emissions, and implementing technology policies that include nonprice regulation, such as efficiency standards, regulation, or targeted R&D policies at different stages of innovation.^34^ However, there is currently a lack of systematic assessment not only in terms of subsequent evidence‐based analysis of different policy instruments, but also of their political feasibility and impact of their distribution within each country.

Furthermore, as energy demand options are neglected in most technoeconomic model scenarios,^35^ many policy options are systematically ignored. Energy demand and location‐specific solutions are likely to be required to achieve sector‐specific targets, as has been shown for the transport sector.^36^ Lifestyle changes, such as diet shifts from meat to vegetarian,^37^ can possibly outperform technological solutions in mitigating emissions in the agricultural sector.^38^ Creutzig et al.^39^ show that both infrastructure provision and nonmonetary incentives emerge as crucial components of comprehensive climate policies, in addition to carbon pricing.

It is the task of innovative research to determine promising policy portfolios for climate change mitigation at global, national, and local scales. However, these tremendous changes cannot be driven by research or policymakers alone. Additionally, it needs initiatives by industry and business to stimulate the required transformation.

## 5 Conclusion

While the 1.5 °C target establishes a limit for what constitutes “dangerous climate change,” the CO_2_ budget for this target is almost exhausted; the attainability of the 1.5 °C target is in jeopardy. The political move toward 1.5 °C highlights the extremely tight budgetary constraints for achieving such a target and pre‐empts a similar debate surrounding the 2 °C target. The controversial discussions on negative emissions are not new, but the growing attention in the political and public arena helps raise awareness on the divergence of action and ambition of this topic. With a rising focus on solutions, this awareness should translate into immediate action.^40^

Rapid decarbonization can be achieved with simultaneous investments in renewable energy technologies, energy demand solutions, and negative emission technologies. We urgently need to work out how to link the climate and development agenda and prevent a buildup of coal power causing lock‐in effects and consuming the remaining carbon budget. We know what to do. Now, we need to find a way to do it.
